# Arginine Relieves the Inflammatory Response and Enhances the Casein Expression in Bovine Mammary Epithelial Cells Induced by Lipopolysaccharide

**DOI:** 10.1155/2016/9618795

**Published:** 2016-03-23

**Authors:** Tianyou Wu, Chao Wang, Luoyang Ding, Yizhao Shen, Huihui Cui, Mengzhi Wang, Hongrong Wang

**Affiliations:** ^1^Laboratory of Metabolic Manipulation of Herbivorous Animal Nutrition, College of Animal Science and Technology, Yangzhou University, Yangzhou 225009, China; ^2^Cell Signaling Group, School of Pathology and Laboratory Medicine, The University of Western Australia, M Block QEII Medical Center, Monash Avenue, Nedlands, WA 6009, Australia

## Abstract

As one of functional active amino acids, L-arginine holds a key position in immunity. However, the mechanism that arginine modulates cow mammary inflammatory response in ruminant is unclear. Therefore, this study was conducted to investigate the effects of L-arginine on inflammatory response and casein expression after challenging the bovine mammary epithelial cells (BMECs) with lipopolysaccharide (LPS). The cells were divided into four groups, stimulated with or without LPS (10 *μ*g/mL) and treated with or without arginine (100 *μ*g/mL) for 12 h. The concentration of proinflammatory cytokines, inducible nitric oxide synthase (iNOS), mammalian target of rapamycin (mTOR), and Toll-like receptor 4 (TLR4) signaling pathways as well as the casein was determined. The results showed that arginine reduced the LPS-induced production like IL-1*β*, IL-6, TNF-*α*, and iNOS. Though the expression of NF-*κ*B was attenuated and the mTOR signaling pathway was upregulated, arginine had no effect on TLR4 expression. In addition, our results show that the content of *β*-casein and the total casein were enhanced after arginine was supplemented in LPS-induced BMECs. In conclusion, arginine could relieve the inflammatory reaction induced by LPS and enhance the concentration of *β*-casein and the total casein in bovine mammary epithelial cells.

## 1. Introduction

Today's intensive dairy cow management systems encourage the inclusion of large amounts of cereal grains in the diets of lactating dairy cows to support high milk yield and enhance cost efficiency. However, overfeeding a high grain diet is associated with subacute rumen acidosis (SARA) which is characterized by rapid and prolonged decrease in ruminal pH and the release of lipopolysaccharide (LPS) due to the increase in lyses of gram-negative bacteria cell in the rumen [1–3]. The LPS accumulated in the rumen can translocate into the peripheral blood circulation and invade the bovine mammary tissue after breaking the Milk-Blood Barrier [4]. Once the LPS entered in the udder, proinflammatory cytokines such as IL-1*β*, IL-6, and TNF-*α* would be excessively produced. Consequentially, local mammary inflammation events were induced via LPS/TLR4 signaling pathways [5–7]. Indeed, normal milk secretion from mammary epithelial cells was disrupted, and the casein expression was also declined [8, 9].

It is widely accepted that arginine serves as a functional amino acid [10]. Except for protein synthesis, arginine holds a key position in both innate and acquired immunity as well. It is the sole substrate for production of nitric oxide (NO) and the precursor for synthesis of polyamines, proline, and agmatine [11] which are important immune molecules. Arginine exerts its immunologic function through regulating the functional activity, proliferation, and apoptosis of immune cells [12]. Additionally, emerging evidence showed that arginine boosts immune function via regulation of mTOR signal pathways [13, 14]. Investigations from monogastric animals and poultry also have indicated that dietary supplementation or intravenous administration of arginine has a positive effect on inflammation and immune response induced by LPS. Zhu et al. claimed that arginine supplementation protected and enhanced weaned pigs' intestinal mucosal immune barrier function and maintains intestinal integrity after* E. coli* LPS challenged [15]. Moreover, Tan et al. found that diet supplementation with 1.42% arginine attenuated the overexpression of proinflammatory cytokines in broiler chickens [16]. However, little research has been conducted in the dairy cattle.

Bovine mammary epithelial cells (BMECs) are the main sites for milk protein synthesis and secretion. They are the first line of defense against bacteria pathogenicity as well [17]. Our previous in vitro studies showed that arginine increased the synthesis of casein in BMECs through activation of mammalian targets of rapamycin (mTOR) and Janus Kinase Signal Transducers 2 and activators of transcription 5 signaling pathways [18]. The objective of this study is to explore whether arginine can relieve the inflammatory response and enhance the expression of casein in BMECs induced by LPS.

## 2. Materials and Methods

### 2.1. Cells Isolation and Culture

All procedures including animals in our study were approved by the Yangzhou University Animal Care and Use Committee of Jiangsu Province, China (SCXK, protocol #2012-0004). Three multiparous dairy cows (513.67 ± 13.05 Kg, day  200.33 ± 5.86  of lactation) were obtained from the Experimental Farm of Yangzhou University. After milking in the morning, tissue sample was taken by biopsy [19]. The sample was placed in DMEM/F12 (1 : 1) medium supplemented with the antibiotic-antimycotic mix (Sigma-A5955) and rinsed several times with PBS including 100 IU/mL mycillin to make sure there was no milk and blood contained. The tissue was then minced to 1 mm^3^ pieces and digested by 0.5% (w/v) collagenase II at 38°C for 3 h. Epithelial cells were obtained after being filtered through a 74 *μ*m strainer and centrifuged for 5 min at 1811 g. These cells were suspended in 5 mL growth medium (DMEM/F12 with 10% FBS, 100 IU/mL mycillin, 2.5 *μ*g/mL amphotericin B, 1 *μ*g/mL prolactin, 1 *μ*g/mL insulin-transferrin, 0.5 *µ*g/mL hydrocortisone, and 10 ng/mL bovine epithelial growth factor) and then were incubated at 38°C in 5% CO_2_ in a 25-square-centimeter plastic culture flask. The growth media were replaced every 48 h. Cells were digested by 0.25% trypsin and 0.1% EDTA-2Na for different times (firstly for 3 minutes and then 5 minutes) to purify the primary epithelial cells when cell number was multiplied and reached 90% confluence. The identification of pure mammary epithelial cells was carried out according to the methods described in our previous works [18]. The identified mammary epithelial cells were used for this study.

### 2.2. Experimental Design and Treatment

The 2nd generation mammary epithelial cells were seeded in 6-well culture plates at the density of 10^6^ per hole for gene expression analysis and seeded in 10 cm cell culture dish at 10^7^ cells per dish for protein concentration analysis. The cells were cultured at 38°C with 5% CO_2_ in humidified incubator. The growth media were changed with DMEM/F12 medium when cells were adherent to the vessel surface. Through a 16 h incubation, cells were divided into four groups and the medium was replaced with different medium as follows:

1. Control, DMEM/F12 medium;
2. Arginine treatment, DMEM/F12 medium supplemented with 100 *μ*g/mL L-arginine;
3. LPS treatment, DMEM/F12 medium supplemented with 10 *μ*g/mL LPS from* E. coli* 055:B5 (Sigma-Aldrich, number L2880);
4. LPS with arginine treatment, DMEM/F12 medium supplemented with both 10 *μ*g/mL LPS and 100 *μ*g/mL L-arginine.

### 2.3. RNA Extraction and Gene mRNA Expression Analysis

After culturing for 12 hours, the total RNA was extracted with TRIzol (Invitrogen) reagent and its quantity and integrity were evaluated with a NanoDrop 1000 spectrophotometer (NanoDrop Technologies, Wilmington, DE, USA). RNA samples were reverse-transcribed using PrimeScript 1st Strand cDNA Synthesis Kit (TaKaRa Code: D6110A, TaKaRa Biotechnology (Dalian) Co., Ltd., Dalian, China) according to the manufacturer's instructions. The real-time PCR was performed with a Bio-Rad IQ5 Real-Time PCR (Bio-Rad Laboratories, Inc., Hercules, CA, USA), using SYBR Premix Ex Taq II Kit (TaKaRa Code: DRR081A, TaKaRa Biotechnology (Dalian) Co., Ltd., Dalian, China). The protocol in detail was described in our previous works [18]. Primers for target and internal reference gene (*β*-actin) were designed with Primer 5.0 and synthesized by Shanghai Sangon Biological Engineering Technology & Services Co., Ltd. (China). The information of primers was displayed in Table 1.

### 2.4. Protein Isolation and Total Protein Concentration Determination

After 12 h incubation with challenge medium, total protein was extracted using RIPA lysis buffer (Beyotime, number P0013B, Beyotime Biotechnology, Shanghai, China) which consisted of a 1 : 100 dilution of phenylmethylsulfonyl fluoride (Sigma) and cocktail (Sigma, number P8340). The concentrations of the total protein were determined with BCA assay kits (Beyotime, number P0010).

### 2.5. Western Blot Analysis

Protein sample was boiled at 100°C for 5 min; 30 *μ*g of total protein per lane was resolved by SDS-PAGE and then transferred to polyvinylidene difluoride (PVDF) membrane (Pall Corporation, Port Washington, NY, USA; # 66543) by using the wet transfer TransBlot assembly (Bio-Rad). Membranes were blocked in Tris-buffered saline (TBS-T; 50 mM Tris, pH 7.6, 150 mM NaCl, and 1% Tween 20) which contains 5% (w/v) Bovine Serum Albumin (BSA) for 2 h at room temperature with gentle agitation. The membranes were then incubated in TBS-TV (TBS-T; 50 mM Tris, pH 7.4, 150 mM NaCl, 1% Tween 20, and 100 mM sodium vanadate) with 5% BSA containing antibodies to *β*-actin (Beyotime, number AA128), TLR4 (Novus Biologicals, number NBP2-24538), NF-*κ*B p65 (CST, #4764), p-NF-*κ*B p65 (CST, #3033), PI3K p85 (CST, #4292), p-PI3K p85 (CST, #4228), AKT (CST, #9272), p-AKT (Ser473) (CST, #4060), mTOR (CST, #2972), and p-mTOR (Ser2448) (CST, #5536) with gentle agitation at 4°C overnight. After incubating with primary antibody, the membranes were washed and incubated with HRP-conjugated secondary antibodies (Beyotime, number A0208) in TBS-TV for 1 h at room temperature. The bolts were washed and then developed with Super ECL Plus reagent (Apply Technologies Inc., number P1010, Beijing, China). The *β*-actin, a housekeeping gene, was used as a positive loading control. The images were captured using a FluorChem HD2 (Protein Simple, USA). The intensities of the bands were measured with Image-ProPlus 6.0 software.

### 2.6. Cytokine Assay

Concentrations of IL-1*β*, IL-6, and TNF-*α* from cellular culture supernatants were determined by Enzyme Immunometric Assay (ELISA) kits (from R&D Systems, #DY401, DY8190, MTA00; assay length was 15.6–1000 pg/mL, 15.6–1000 pg/mL, 10.9–700 pg/mL). The examination steps in detail were carried out as the manufacturer's protocol described. The optical density of each well was read at 450 nm.

### 2.7. Measurement of Nitric Oxide (NO) Content

Cellular culture supernatants (0.5 mL) were gathered separately at 1 h and 12 h after cells were incubated with challenge medium. The amount of NO secreted in supernatants was determined with the NO detection kit (Beyotime, number A0208). Briefly, 100 *μ*L of supernatants or standard NaNO_2_ was mixed with 100 *μ*L Griess reagent in a 96-well plate and incubated in room temperature for 15 min. NO concentration was determined based on the standard curve by measuring the absorbance at 570 nm on a microplate reader.

### 2.8. Measurement of Casein Content

The content of *α*-, *β*-, and *κ*-casein was measured by ELISA kits (from R&D, Minneapolis, MN, USA, divided by Xinyu Company, Shanghai, China, # CK-E94189B, CK-E94192B, CK-E94193B). Their assay length was 20–320 *μ*g/mL, 25–400 *μ*g/mL, and 12.5–200 *μ*g/mL. The examination steps in detail were carried out as the manufacturer's protocol described. The optical density of each well was read at 450 nm

### 2.9. Statistical Analysis

All data sets of this study were expressed as the mean ± SEM of three independent experiments. One-way analysis of variance (SPSS V16.0 software; SPSS Inc., Chicago, IL) was performed for statistical analysis.  *P* < 0.05  was considered significant difference.

## 3. Results 

### 3.1. Arginine Reduced LPS-Induced Proinflammatory Cytokines Production in BMECs

**Figure 1:**
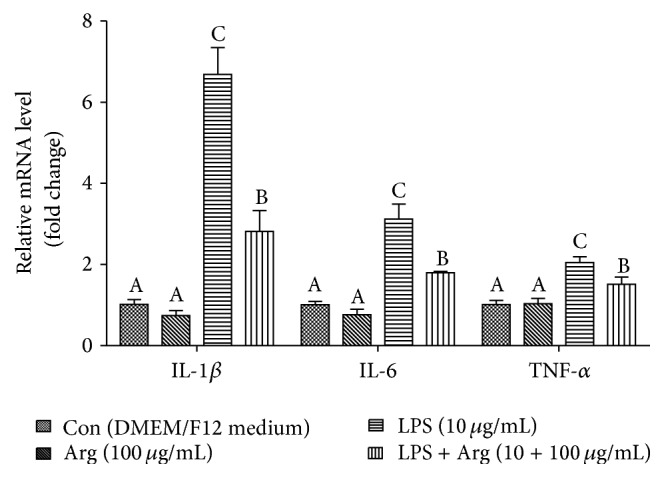
The effect of arginine on LPS-induced cytokines mRNA expression in bovine mammary epithelial cells (BMECs). Pure 2nd generation BMECs were starved for 16 h and then cultured for 12 h in DMEM/F12 medium containing 0 or 10 *μ*g/mL LPS and 0 or 100 *μ*g/mL Arg. The mRNA levels of IL-1*β*, IL-6, and TNF-*α* were analyzed using qPCR. *β*-Actin was used as an internal reference gene. Data shown are means ± SEM of three independent experiments. Means with different letters (A, B, or C) are significantly different (*P* < 0.05) from each other.

Relative mRNA expression levels for IL-1*β*, IL-6, and TNF-*α* are illustrated in Figure 1. In comparison with the contrast quarter, addition of LPS dramatically increased the mRNA abundance of IL-1*β* (6.7-fold higher;  *P* < 0.001), IL-6 (3.1-fold higher;  *P* < 0.001), and TNF-*α* (2.0-fold higher;  *P* < 0.001). And addition of arginine inhibited mRNA levels of IL-1*β* (2.4-fold lower;  *P* < 0.001), IL-6 (1.7-fold lower;  *P* < 0.001), and TNF-*α* (1.4-fold lower;  *P* < 0.05) in cells induced with LPS, compared with LPS quarter. The effect of arginine on the protein expression of IL-1*β*, IL-6, and TNF-*α* in LPS-activated BMECs was similar to that of gene expression (Table 2).

### 3.2. Arginine Inhibited LPS-Induced NF-*κ*B p65 Expression but Had No Effect on TLR4 Expression

**Figure 2:**
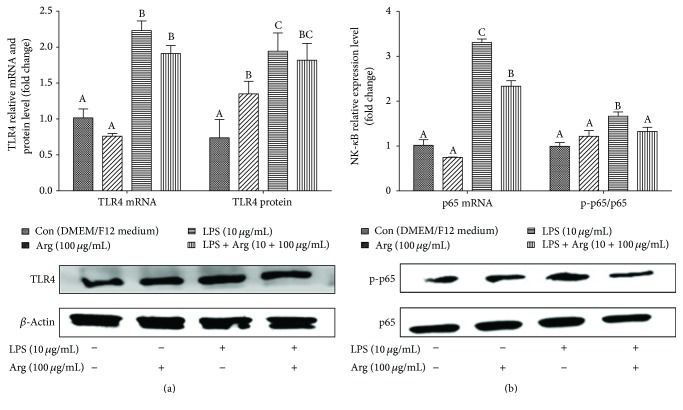
The effect of Arg on LPS-induced TLR4, NF-*κ*B mRNA, and protein expression in BMECs. Pure 2nd generation BMECs were starved for 16 h and then cultured in DMEM/F12 medium containing 0 or 10 *μ*g/mL LPS and 0 or 100 *μ*g/mL Arg. After 12 h, the mRNA levels of TLR4 and NF-*κ*B were analyzed using qPCR. Protein expression was analyzed using western bolt. *β*-Actin was used as an internal reference. Data shown are mean ± SEM of three independent experiments. Means with different letters (A, B, or C) are significantly different (*P* < 0.05) from each other.

As shown in Figure 2, in contrast to control quarter, the levels of TLR-4 and NF-*κ*B p65 gene expression were significantly augmented after BMECs were stimulated with LPS (*P* < 0.01). It was worth to highlight that, in contrast to LPS quarter, supplementation of arginine in LPS-induced BMECs significantly decreased (*P* < 0.05) the NF-*κ*B p65 expression and the level of NF-*κ*B p65 phosphorylation (Figure 2(b)), but it had no effect (*P* > 0.05) on TLR4 expression (Figure 2(a)).

### 3.3. Effects of Arginine on iNOS and NO in LPS-Stimulated BMECs

**Figure 3:**
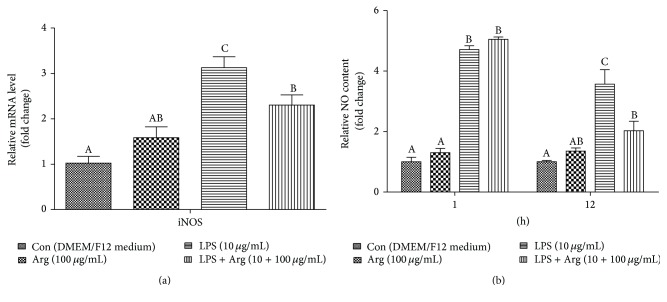
Effects of Arg on iNOS expression in LPS-treated BMECs. Pure 2nd generation BMECs were starved for 16 h and then cultured for 12 h in DMEM/F12 medium containing 0 or 10 *μ*g/mL LPS and 0 or 100 *μ*g/mL Arg. The mRNA levels of iNOS were analyzed using qPCR (a). *β*-Actin was used as an internal reference. 0.5 mL cellular supernatants were gathered at 1 h and 12 h separately after cells were incubated with challenge medium. Then NO content in supernatants was analyzed using NO detection kit (b). Data shown are mean ± SEM of three independent experiments. Means with different letters (A, B, or C) are significantly different (*P* < 0.05) from each other.

The result of iNOS mRNA abundance and NO content is shown in Figure 3. The mRNA level of iNOS in LPS with arginine treatment effectively towering above in the control quarter (*P* < 0.01) and arginine quarter (*P* < 0.01) was lower than that in the LPS quarter (*P* < 0.05). NO content in the LPS quart and LPS with arginine treatment was dramatically higher than (*P* < 0.05) that in the control quarter and arginine quarter both at 1 h and 12 h. To our surprise, NO content in the LPS with arginine treatment did not differ (*P* > 0.05) when compared with LPS quarter at 1 h but was remarkably below (*P* < 0.01) the LPS quarter at 12 h.

### 3.4. Effects of Arginine on PI3K/AKT/mTOR Signaling Pathway in LPS-Stimulated BMECs

**Figure 4:**
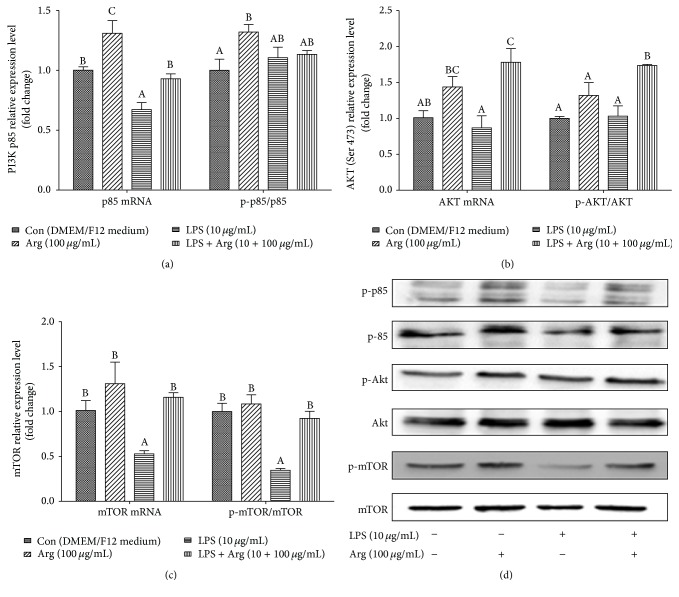
Effect of Arg on the mRNA expression and phosphorylation levels of PI3K/AKT/mTOR signaling pathway in LPS-treated BMECs. Pure 2nd generation BMECs were starved for 16 h and then cultured for 12 h in DMEM/F12 medium containing 0 or 10 *μ*g/mL LPS and 0 or 100 *μ*g/mL Arg. The mRNA levels of PI3K, AKT, and mTOR were analyzed using qPCR. Protein expression and its phosphorylation level were analyzed using western bolt. *β*-Actin was used as an internal reference. Data shown are mean ± SEM of three independent experiments. Means with different letters (A, B, or C) are significantly different (*P* < 0.05) from each other.

The results of the relative gene expression of PI3K, AKT, and mTOR are shown in Figure 4. The results showed that the abundance of PI3K and mTOR gene expression in the LPS quarter was markedly lower (*P* < 0.05) than that in the control quarter, yet the abundance of AKT was not significantly different (*P* > 0.05). Compared with LPS quarter, the mRNA levels of PI3K, mTOR, and AKT in quarter treated with both LPS and arginine were markedly enhanced (*P* < 0.05). We also found that LPS dramatically decreased (*P* < 0.01) the level of p-mTOR/mTOR, but it had no effect on level of p-PI3K/PI3K and p-AKT/AKT (*P* > 0.05). In contrast to LPS quarter, the level of p-AKT/AKT and p-mTOR/mTOR in cells treated with LPS and arginine was notably augmented (*P* < 0.01), but the level of p-PI3K/PI3K was not affected (*P* > 0.05).

### 3.5. Arginine Increased the *β*-Casein and Total Casein Synthesis in LPS-Treated BMECs

The results of the casein concentration are shown in Table 3. The results showed that the protein expression of *β*-casein and total casein in the LPS quarter was significantly lower (*P* < 0.01) than that in the control quarter while *α*-casein had a slight improvement (*P* < 0.1). The levels of *β*-casein and total casein synthesis in quarter of LPS with arginine were markedly higher (*P* < 0.05) than quarter of LPS, yet the *α*-casein and *κ*-casein synthesis had no significant change (*P* > 0.05).

## 4. Discussion

The ruminant digestive tract harbors numerous gram-negative bacteria that are capable of producing lipopolysaccharide (LPS), but the epithelium acts as a barrier to prevent it from entering into the systemic circulation. However, following tract epithelium barrier failure during grain-induced SARA, LPS can potentially be translocated into the interior of the body, for example, into the blood circulation and lymphatic system. Once translocated, LPS interacts with cells and stimulates the production of proinflammatory mediators such as cytokines, and immune response may expand in the mammary gland.

BMECs are the first cells confronted with the bacteria pathogens and are proposed as an important line to defend the bacteria's invasion in the mammary issue [17, 20]. Evidence indicated that a rapid and strong inflammatory response would be induced when BMECs were challenged with LPS [21]. In the present study, the BMECs were challenged with 10 *μ*g/mL LPS [22] to imitate the inflammatory state that rumen LPS invades the BMECs after injuring the Milk-Blood Barrier. Arginine is a semiessential amino acid for animals. It is insufficient during times of physiologic stress such as wound, histological damage, and inflammation. It has been reported that supplement with arginine in the state of pathologic contributes to strengthening the immunity from disease [23, 24]. Thus, a following trial that supplemented LPS-induced BMECs with 100 *μ*g/mL arginine [25] was conducted to investigate the effects of arginine on inflammatory response.

It is well known that LPS-induced inflammation events are characterized by eliciting and releasing a large number of proinflammatory cytokines (such as TNF-*α*, IL-1*β*, and IL-6) in macrophages and epithelial cells [26]. These cytokines have a vital role in promoting and regulating the immune function response to bacterial pathogens [27]. And the expression of them, to a certain extent, reflects the magnitude of inflammation response. In this experiment, we confirmed that the concentrations of IL-1*β*, IL-6, and TNF-*α* were remarkably increased at 12 h after BMECs were stimulated with LPS. At the same time, we found that arginine inhibited the secretion of those cytokines. Similar result had been reported by Tan et al. and Calkins et al. [16, 28]. Moreover, Jiang et al. also found that treatment with 100–300 *μ*g/mL arginine in intestine of juvenile carp completely prevents increase of IL-6 and TNF-*α* and statistically significant decrease of IL-1*β* mRNA expression [25]. This result indicated that arginine would contribute to attenuating the inflammatory response.

For further insight into the possible mechanisms that arginine suppresses the proinflammatory cytokines expression, we investigated the expression level of TLR4 and NF-*κ*B, the vital upstream signaling molecules in regulating the LPS-induced inflammatory response. TLR4, the bacterial LPS receptor, has been shown to be responsible for LPS recognition by cooperating with CD14; it led to the degradation of I*κ*B and liberation of NF-*κ*B via myeloid differentiation factor 88 (MyD88), IL-1R-associated kinase (IRAK) and TNFR-associated factor 6 (TRAF6). Then NF-*κ*B was translocated into the nucleus and induced transcription of proinflammatory genes after it was activated by phosphorylation [29–31]. Thereby inhibition of NF-*κ*B activation has been considered as a critical target and effective approach to curb the inflammatory response [32, 33]. Our study showed that stimulation with LPS augmented the expression of NF-*κ*B p65 and the phosphorylation of NF-*κ*B p65. It prompted that NF-*κ*B p65 was active and redound to proinflammatory expression. These results were concordant with previous research by Kim et al. and Verma et al. [4, 34]. Nevertheless, the elevation of NF-*κ*B p65 expression was completely reversed after supplementing with arginine. It may imply that arginine diminishes the expression of proinflammatory through inhibiting the activation of NF-*κ*B.

Arginine is the sole precursor for the production of NO by one of the three NO synthases (NOSs). Under normal physiological condition, low concentrations of NO are generated by eNOS (endothelial NOS) and nNOS (neuronal NOS) isoforms. But, in pathological conditions (virus and bacterial infection), high-level NO is synthesized by iNOS to promote the proliferation of immunologic cells and to defend against the pathogenic bacteria. Despite the fact that the high NO concentration in the early stages of inflammation boosts the immune function and inhibits the phosphorylation of NF-*κ*B [12], it also would lead to uncontrolled tissue injury and may cause inflammatory diseases [35–37]. In this experiment, we gauged the inducible NOS (iNOS) mRNA expression level and the NO content in cell supernatant. Our investigation shows that LPS challenge upregulated the gene expression of iNOS and elevated the contention of NO, which was consistent with previous research [38, 39]. But out of our expectation, arginine administration inhibits the iNOS mRNA expression and declined the NO content at 12 h in LPS-induced BMECs. Similarly, Xue found that arginine accession increased the activity of eNOS but not iNOS after immature myocardial ischemia-reperfusion injury [40]. Moreover, Colasanti et al. suggested that both sodium nitroprusside (SNP, NO donor) and authentic NO solution are able to inhibit LPS-induced iNOS mRNA expression [41]. That may give evidence to support our observation. In our study, the result that arginine administration downregulated the iNOS mRNA expression and the decline of NO content at 12 h suggested that arginine may be able to alleviate the inflammatory injury after LPS-induced acute inflammation.

The PI3K/AKT/mTOR pathways have been recognized to be critically involved in cell metabolism, growth, survival, and vesicular transport for a long time [42, 43]. Recently, a growing body of evidence indicated that this pathway also participated in the innate and adaptive immunity modulation [44–46]. Many known receptors such as cytokine receptors, insulin, insulin-like growth factor I (IGF-1) receptor, and also Toll-like receptors are able to activate the PI3K/AKT/mTOR pathway by enhancing the phosphorylation of PI3K and subsequently AKT and mTOR [47, 48]. Present data in this study showed that LPS treatment could alter the expression of PI3K/AKT/mTOR pathway (reduced the mRNA expression of PI3K and mTOR, decreased the level of p-mTOR/mTOR). These findings indicated that the PI3K/AKT/mTOR pathway correlates closely to LPS-induced epithelial cells inflammation events. We also found that arginine administration enhanced the mRNA and its phosphorylation expression of AKT and mTOR (Figure 4). Mendes et al. suggest that the PI3K/AKT/mTOR could negatively regulate the effects of inflammatory response induced by LPS through blocking NF-*κ*B transactivation in vitro model of murine macrophage [49]. Guha and Mackman and Zhang et al. indicated inhibition of PI3K/AKT signaling in human monocytic THP-1 cells enhancing NF-*κ*B activation [50, 51]. However, the paradoxical results were reported by Xie et al., Lorne et al., and Fortin et al. [46, 52, 53]. They claimed that inhibitors of PI3K and mTOR could remarkably suppress the secretion of cytokines by monocytes and macrophages and human neutrophils. Different cell types and treatments may answer for those paradoxical results. Up to now, there has no direct proof to testify how PI3K/AKT/mTOR pathway accommodates the LPS-induced inflammation in mammary epithelial cells. Therefore, further research is needed to address a series of mechanistic questions in order to increase understanding of arginine involved in the LPS-induced inflammation modulation.

Another purpose of current study is to explore the effects of arginine on casein synthesis in LPS-stimulated BMECs. Bovine lactoprotein is an important component of the human diet. Casein is the major ingredient of milk protein, approximately occupying 80 percent of the total lactoprotein, with *α*-casein (*α*-CN) comprising 45–55%, *β*-casein (*β*-CN) comprising 25–35%, and *κ*-casein (*κ*-CN) comprising 8–15%. The content of casein synthesis is often deemed to the mark of capacity of synthesizing protein and condition of cell function. LPS in bovines' rumen translated to mammary gland after it suffered from SARA, mastitis would be induced, and a series of adverse events would occur, one of which is reduction of casein synthesis [9]. In the current study, we imitated the state that rumen LPS invade the mammary gland by establishing the inflammation model with LPS stimulating the BMECs. We found that LPS markedly declined the *β*-casein and the total casein content, but also slightly played down the content of *α*-CN and *κ*-CN in spite of having no significant difference. Parallel results were reported by Hinz et al. and Schmitz et al. [54, 55]. We also found that arginine supplementation was able to reverse the tendency of LPS-caused reduction of casein synthesis. Finally, current study demonstrated that arginine is able to enhance the expression of *β*-casein and the total casein.

## 5. Conclusion

**Figure 5:**
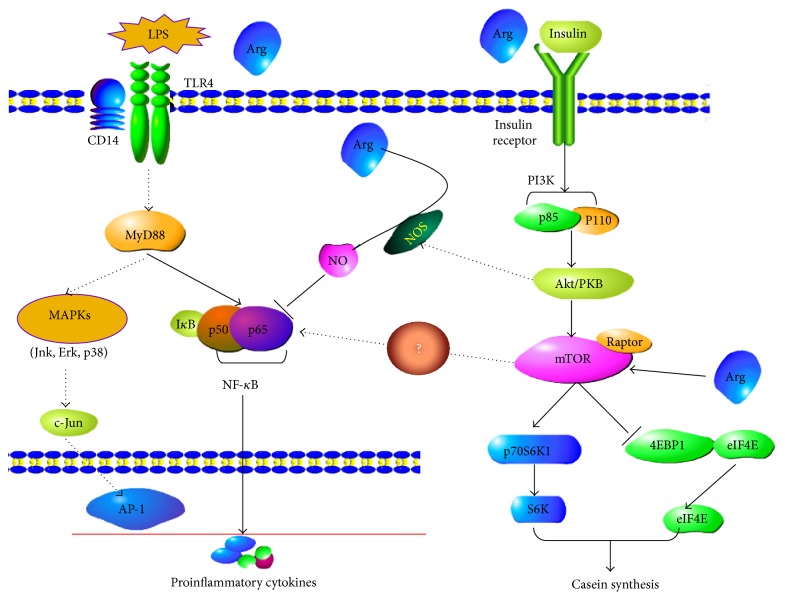
The potential mechanism that arginine relieves the inflammatory response and enhances the casein expression in bovine mammary epithelial cells induced by lipopolysaccharide.

In summary, the results from the present experiment implied that arginine effectively attenuated LPS-induced bovine mammary epithelial cells inflammatory response by inhibiting NF-*κ*B signaling pathways. Additionally, arginine may be involved in Arg/NO and PI3K/AKT/mTOR pathway. Further, arginine was also able to enhance the *β*-casein and the total casein expression in LPS-induced bovine mammary epithelial cells (Figure 5).

## Figures and Tables

**Table 1:** Primers utilized for quantitative real-time PCR analysis.

Gene	Forward primer	Reverse primer	Product size (bp)	Amplification efficiency
*β*-Actin	CCTGCGGCATTCACGAAACTAC	ACTCCTGCTTGCTGATCCACATC	273	96.75%
TLR-4	CGGCACAGACAGAGGGTTAT	GGTCCAGCATCTTGGTTGAT	240	104.15%
NF-*κ*B	CCCTTCCAAGTTCCCATAGAA	CTCCCAGAGTTCCGATTCAC	197	93.55%
iNOS	GGACTTGGCTACGGAACTGG	GGTGAAGCGTGTCTTGGAAA	257	89.21%
IL-1*β*	CCTCCGACGAGTTTCTGTGT	AAAGCTCATGCAGAACACCA	161	93.51%
IL-6	GCTCTCATTAAGCGCATGGT	AGCAAATCGCCTGATTGAAC	172	95.38%
TNF-*α*	CCTGCCAATGTTTCCAGACT	GGCAGCCCTTAGTTTGTGTC	183	99.71%
PI3K	GATGCTACCTTACGGCTGCT	CGGCACAGGATAGGGTAAAC	215	94.39%
AKT	CACCATTACGCCACCTGAC	CACTCAAACGCATCCAGAAA	233	101.27%
mTOR	CATGTGCGAACACAGCAACA	TGCCTTTCACGTTCCTCTCC	149	94.59%

**Table 2:** Effects of Arginine on protein expression of cytokines in LPS-treated BMECs.

	Con (DMEM/F12 medium)	Arg (100 *µ*g/mL)	LPS (10 *µ*g/mL)	Arg + LPS (100 *µ*g/mL + 10 *µ*g/mL)
IL-1*β* (pg/mL)	94.51 ± 0.36^a^	116.91 ± 1.86^a^	310.89 ± 4.84^c^	243.15 ± 16.12^b^
IL-6 (pg/mL)	33.61 ± 0.74^a^	34.89 ± 1.36^a^	103.68 ± 0.92^c^	81.70 ± 0.87^b^
TNF-*α* (pg/mL)	36.78 ± 1.04^a^	38.61 ± 0.56^a^	65.98 ± 1.13^c^	60.36 ± 3.92^b^

Values are expressed as mean ± SEM, *n* = 3. Mean with different letters differ (a, b, or c) are significantly different (*P* < 0.05) from each other.

**Table 3:** Effects of arginine on casein synthesis in LPS-treated BMECs.

	Con (DMEM/F12 medium)	Arg (100 *µ*g/mL)	LPS (10 *µ*g/mL)	Arg + LPS (100 *µ*g/mL + 10 *µ*g/mL)
*α*-Casein (*µ*g/mL)	164.91 ± 2.89^a^	179.80 ± 4.33^b^	150.49 ± 6.70^a^	155.90 ± 1.73^a^
*β*-Casein (*µ*g/mL)	75.10 ± 2.41^bc^	82.29 ± 5.87^c^	52.25 ± 3.22^a^	67.38 ± 1.62^b^
*κ*-Casein (*µ*g/mL)	24.66 ± 4.82	27.07 ± 5.02	19.89 ± 7.60	21.77 ± 2.86
Total casein^1^ (*µ*g/mL)	264.67 ± 0.89^b^	289.16 ± 1.39^c^	222.62 ± 11.03^a^	255.37 ± 7.83^b^

^1^Total casein = *α*-casein + *β*-casein + *κ*-casein.

Values are expressed as mean ± SEM, *n* = 3. Mean with different letters differ (a, b, or c) are significantly different (*P* < 0.05) from each other.
